# Employees and the National Insurance Act

**Published:** 1912-04-13

**Authors:** 


### NATIONAL INSURANCE ACT DEPARTMENT.

### EMPLOYEES AND THE NATIONAL INSURANCE ACT.

### VI.—Benefits Receivable under the Act—(*continued*).

The benefits with which we have dealt in our last three issues have been styled " Minimum Benefits " by some authorities. They are not so described in the Act itself, and we have preferred to call them the " standard benefits." The Act provides for reductions in the rates of benefit from the outset in the case of persons over fifty years of age, and this, in itself, is sufficient justification for the rejection of the word " minimum " as a correct description of the benefits in question. Further than this it may be necessary in three years' time, after the first valuations have been made, for some societies to reduce the rates of benefit below those provided by the Act. This will occur when the valuation discloses a deficiency, which cannot be made good in any other way. In these circumstances we hold that the term " standard " is a better description than " minimum."

Additional Benefits.

The word " minimum " was apparently introduced because the Act provides a number of " additional " benefits, which in certain circumstances may apply. These additional benefits can be made available in two ways. It is possible that they may be brought into operation at the outset, or they may be deferred until the first or some subsequent valuation discloses a surplus. In the first case the " additional " benefit will partake of the nature of an alternative, being in substitution, in whole or in part, for sickness and disablement benefits, or either of them. Only in the second case—namely, where there is an available surplus—will the " additional " benefit be really in addition to the standard benefits. It is conceivable that there are, or that there may be, certain classes of persons within the description of employed contributors to whom the sickness and disablement benefits present no attraction, in fact such benefits may be entirely superfluous. A much discussed case whilst the Bill was before the House was that of the domestic servant, and we may take this as an example. It has been customary in this class of employment for full wages to be paid, though the servant was unable to fulfil her duties by reason of illness. The payment of sickness benefit where full wages are being drawn is not only unnecessary but undesirable, and thus we have a case in which it is eminently to the advantage of the insured person to be able to substitute an alternative benefit. The alternative benefit will have to be selected from the list of additional benefits allowed by the Act.

It must be clearly understood, however, that these additional benefits cannot be obtained by a deposit contributor. They are only available for members of approved societies, and, in order that they may be obtained, it will be necessary for the society to draw up a scheme and submit it for the approval of the Insurance Commissioners. For the protection of the insured persons it is provided that no such scheme shall be confirmed by the Commissioners unless they are satisfied that the value of the alternative benefit or benefits is equivalent to that relinquished. It will also be necessary for the society to show good reason for the suggested substitution as applied to the society or to the class of members intended to come under the scheme.

If an insured person desires to avail himself of one or other of the additional benefits in place of sickness benefit his first step will be to find a society with a scheme of benefits to meet his requirements. Unfortunately it is not yet possible to indicate the precise lines the societies are taking in the formation of their schemes. It is known that such schemes are in course of preparation, but the calculations of value upon which they are to be based, take time to make, and the calculations are dependent upon the reserves to be credited to the societies at the outset in consideration of members joining at ages above sixteen. These reserves are not yet published, and in consequence some weeks will elapse before precise information can be furnished in regard to details.

In the meantime we may, however, consider the general character of the additional benefits, some one or more of which it may be desired to obtain. The list is set out in the Fourth Schedule, Part II., and contains in all fourteen benefits. They are of a very varied character, but there is one notable omission—namely, benefits payable on death. This omission is not by way of oversight but by express exclusion. At first sight it may appear to be difficult to account for this exclusion because the benefits in general follow the usual practice of the existing Friendly Societies, and most of these societies include among their benefits payments on the death of a member and on the death of his wife. These death benefits are, as a rule, of small amount, being about sufficient to meet the funeral expenses.

Reason for Exclusion of Death Benefits.

The Friendly Societies, however, are not the only bodies concerned in the provision of funeral benefits. The large Industrial Assurance Companies—the Prudential, Pearl, Britannic, Salvation Army, and others—and the Collecting Friendly Societies, of which the Liverpool Victoria Legal and the Royal Liver are examples, have by their widely extended agency systems brought within the reach of all classes of the community the means of providing for payments on death. This has been done without any State subvention, and no material gain would have accrued to the wage-earning classes by the provision of further facilities in this respect. It has already been pointed out in an earlier issue that, in spite of the excellent work done by the Friendly Societies, they had failed, not entirely through any fault of their own making, to appeal to large numbers of those to whom insurance against sickness is really essential. If in the endeavour to deal with health insurance on a National basis, the Friendly Societies had been allowed to apply any portion of the State subsidy in the provision of death benefits, an injustice would have been inflicted on the Industrial Companies and Collecting Societies. Hence there is a valid reason for the exclusion from the Act of any benefits of this particular type. The advisability—we had almost said the necessity—of making provision for funeral expenses and for something in addition whereby dependants may be relieved of anxiety and the stress of poverty after the demise of the bread-winner will remain as important after the Act comes into operation as it has ever been. Those who are already assured will be wise to maintain their policies, whilst those who have not already effected an assurance should do so as early as possible, but it must be understood that the payment of premiums will remain voluntary as heretofore, and no subsidy from public funds can be expected now, or in the future, towards benefits payable on death.

The first additional benefit allowed is medical treatment and attendance for any persons dependent upon the labour of a member. It will be remembered in this connection that medical benefit is the first of those in the standard list. So far as the standard benefit is concerned, it only applies to the insured person, but under the additional scheme it may be extended to those who are dependent upon the member. This will, of course, be a very valuable benefit, but it is to be feared that it will prove too costly to be included in any initial scheme, quite apart from the difficulty that is likely to arise between the societies on the one hand and the medical profession on the other in the arrangement of terms. In course of time, after the Act has been given a fair trial, and the administration of the standard medical benefit has been tested by experience, it may be possible to secure the extension to dependants. Owing to the fact that the number of dependants will vary considerably per member, it would seem that any such scheme will have to apply to the society as a whole, and could not be made available at the choice or option of the individual member.

Realising the importance of the care of the teeth as a means of securing and maintaining good health, the framers of the Act have provided that another form in which additional benefit may be taken is the payment of the whole or any part of the cost of dental treatment. The greater attention that has been paid to the teeth during recent years, particularly as regards children at school, has doubtless effected considerable improvement, but much yet remains to be done in the education of the masses as to the care of the teeth, and it is eminently satisfactory that this way of spending any part of the sickness or disablement allowances that may not be required, or of applying surplus in the same manner, is allowed by the Act. As to how soon it will be found possible for the societies to take advantage of the provision is a matter of doubt, but before joining a society it would be well if inquiries were made to elicit information as to whether any such benefit may be expected in the future.

### Increase of Benefits.

The next five clauses in the Schedule of Additional Benefits are in the nature of increase in the rates as provided by the standard. It is therefore reasonable to suppose that neither of these five clauses can be taken to apply as substituted benefits in place of the standard sickness and disablement allowances that it may be desired to relinquish. These clauses will probably be the ones of which greatest advantage will be taken when the time comes for a distribution of surplus. In order to conduce to the payment of the increase of sickness or disablement benefit amongst those to whom such increase is most necessary, it will be possible for the societies to frame their schemes in such a way that the increased benefit shall only be paid to members who have children, or a specified number of children dependent upon them. This provision is a clear indication of the amount of foresight displayed by the framers of the Act in their endeavour to make the measure as effective as possible in dispensing relief where it is most required. Not only may the rates of sickness benefit be increased, but either in addition, or as an alternative, to such increase it may be decided to make the allowance accrue as from the first, second, or third day after the commencement of the disease or disablement. It will be remembered, of course, that the standard sickness benefit only operates as from the fourth day of sickness, and we commented in a previous issue on the departure in this respect from the existing practice of most societies. We also referred in an earlier article to the rule frequently to be met with amongst Friendly Societies, whereby the member is allowed to undertake certain forms of light employment whilst in the receipt of benefit. The standard sickness and disablement benefits are, however, only payable whilst the member is "incapable of work," and this phrase is apparently intended to be construed as meaning incapable of doing any work. Thus we have as a concession in the form of an additional benefit, which may be adopted by any society if it obtains sanction for its scheme, that disablement allowance can be paid to its members, though not totally incapable of work. Similarly it will be possible for the society to include the payment of allowances to members while convalescent from some disease or disablement in respect of which the standard benefit has been payable.

The last of the clauses dealing with increase of benefits allows the increase of maternity benefit. Time alone can show whether it is desirable for an increase in this direction.

The clauses dealt with in this article are the first seven in the Schedule of Additional Benefits; we shall return to the consideration of the remaining clauses next week.

Note: Questions and Correspondence on all doubtful points are invited, and should be addressed to the Insurance Editor, The Hospital, 28 Southampton Street, Strand.

